# Bis{μ-2-[3-carboxyl­atometh­yl-4-(phenyl­sulfan­yl)phen­yl]propano­ato-κ^4^ *O*,*O*′:*O*′′,*O*′′′}bis­[(2,2′-bipyridine-κ^2^ *N*,*N*′)cadmium]

**DOI:** 10.1107/S1600536812013049

**Published:** 2012-03-31

**Authors:** Long Li, Yu-Qiu Ding, Kai-Sheng Diao

**Affiliations:** a School of Chemistry and Chemical Engineering, Guangxi University for Nationalities, Nanning 530006, People’s Republic of China

## Abstract

In the title complex, [Cd_2_(C_17_H_14_O_4_S)_2_(C_10_H_8_N_2_)_2_], which was hydro­thermally synthesized, the Cd^II^ cation is hexa­coordinated in a distorted octa­hedral geometry by two N atoms from a 2,2′-bipyridine ligand and by four O atoms from two different 2-[3-carboxyl­atometh­yl-4-(phenyl­sulfan­yl)phen­yl]propano­ate ligands, forming a cyclic dimetallic complex.

## Related literature

For reviews of metal-organic network solids, see: Batten & Robson (1998 ▶); Lu (2003 ▶); Moulton & Zaworotko (2001 ▶); Pan *et al.* (2004 ▶). For the synthesis and structure of helical Cd complexes with related ligands, see: Wang *et al.* (2004 ▶).
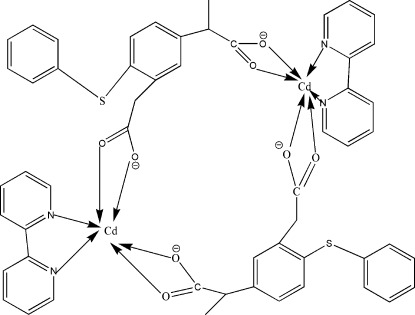


## Experimental

### 

#### Crystal data

[Cd_2_(C_17_H_14_O_4_S)_2_(C_10_H_8_N_2_)_2_]
*M* *_r_* = 1165.85
Monoclinic, 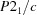

*a* = 13.567 (3) Å
*b* = 11.572 (3) Å
*c* = 15.595 (4) Å
β = 92.540 (3)°
*V* = 2446.2 (10) Å^3^
*Z* = 2
Mo *K*α radiation
μ = 1.02 mm^−1^
*T* = 296 K
0.35 × 0.34 × 0.32 mm

#### Data collection

Bruker SMART BREEZE CCD area-detector diffractometer
Absorption correction: multi-scan (*SADABS*; Sheldrick, 1996 ▶) *T*_min_ = 0.718, *T*_max_ = 0.737
14971 measured reflections
5706 independent reflections
4170 reflections with *I* > 2σ(*I*)
*R*_int_ = 0.042

#### Refinement

*R*[*F* ^2^ > 2σ(*F* ^2^)] = 0.036
*wR*(*F* ^2^) = 0.090
*S* = 1.04
5706 reflections
329 parameters
3 restraints
Δρ_max_ = 0.59 e Å^−3^
Δρ_min_ = −0.67 e Å^−3^

### 

Data collection: *APEX2* (Bruker, 2009 ▶); cell refinement: *SAINT* (Bruker, 2009 ▶); data reduction: *SAINT*; program(s) used to solve structure: *SHELXS97* (Sheldrick, 2008 ▶); program(s) used to refine structure: *SHELXL97* (Sheldrick, 2008 ▶); molecular graphics: *SHELXTL* (Sheldrick, 2008 ▶); software used to prepare material for publication: *SHELXTL*.

## Supplementary Material

Crystal structure: contains datablock(s) I, global. DOI: 10.1107/S1600536812013049/mw2060sup1.cif

Structure factors: contains datablock(s) I. DOI: 10.1107/S1600536812013049/mw2060Isup2.hkl

Additional supplementary materials:  crystallographic information; 3D view; checkCIF report

## Figures and Tables

**Fig. 1.**
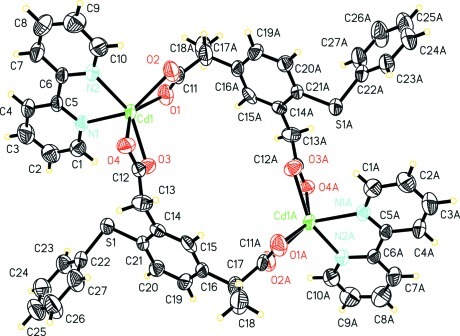
The molecular structure of (I) showing 50% probability displacement ellipsoids and the atom-numbering scheme.

**Fig. 2.**
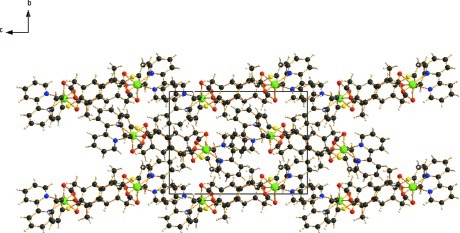
The molecular packing diagram for the crystal of (I).

## supplementary crystallographic information

### Comment

The design and synthesis of coordination polymers is of great interest due to their intriguing topologic architecture and significant application in many fields (Pan *et al.*, 2004; Batten *et al.*, 1998). Among the variety of organic molecules acting as linkers in the design of supramolecular networks, heterocyclic N rings and polycarboxylates are the most widely used ligands due to their rigidity in structure and flexibility in coordination modes (Moulton *et al.*, 2001; Wang *et al.*, 2004; Lu *et al.*, 2003). In this work we report a cyclic Cd^II^ coordination complex, (I), constructed from 5-(1-carboxyethyl)-2-(phenylthio)phenylacetic acid and 2,2'-bipyridine (Fig. 1). The O—Cd bond distances range from 2.277 (6) to 2.416 (6) Å and the Cd—N bond distances are 2.311 (3) and 2.320 (3) Å. The dihedral angle between two benzene rings in the same *L* ligand is 78.73 (6)° and the C—S—C angle is 102.11 (6)°. It is to be noted that both carboxylates of *L* are bidentate but asymmetrically coordinated with the Cd1—O1 and Cd1—O2 bond distance of 2.276 (3) and 2.415 (3) Å, respectively while the Cd1—O3 and Cd1—O4 distances are 2.345 (3) and 2.309 (3) Å. These differences can likely be attibuted to packing considerations since of the four carboxylate oxygen atoms, only O1 is not involved with C—H···O hydrogen bonding interactions. The self-assembly of the metal with 5-(1-carboxyethyl)-2-(phenylthio)phenylacetic acid and 2,2'-bipyridine links the Cd ions into a cyclic dimer (Fig. 1). Weak π-π stacking interactions between pyridine rings from different bipy ligands (interplanar spacing = 3.708 (4) Å, dihedral angle between planes = 1.07 (4)°) as well as a number of short (C—H···*X* (*X* = O, N)) contacts generate a 3-D structure (Fig. 2).

### Experimental

H_2_L (0.5 mmol) and 2,2'-bipyridine (0.5 mmol) were dissolved in a mixture of 5 ml of ethanol and 15 ml of H_2_O to which an aqueous solution of sodium hydroxide was added dropwise with stirring to adjust the pH to 6. After addition of 10 ml of an aqueous solution of cadmium chloride (0.5 mmol) the mixture was heated at 403 K for 3 days. After cooling to room temperature, the reaction solution was filtered to remove a small quantity of white precipitate. Slow evaporation of the filtrate at room temperature over three days produced X-ray quality colorless block-shaped single crystals.

### Refinement

H atoms were positioned geometrically and refined as riding atoms with C—H = 0.93 Å and *U*_iso_(H) = 1.2*U*_eq_(C) for aromatic H atoms, C—H = 0.97 Å and *U*_iso_(H) = 1.2*U*_eq_(C)for methylene H atoms, and C—H = 0.96 Å and *U*_iso_(H) = 1.5*U*_eq_(C) for methyl H atoms. Atoms C17 and C18 are disordered over two distinct sites in a 77:23 ratio. The two components of this disorder were refined with restraints to make their geometries similar.

### Crystal data

**Table:**

[Cd_2_(C_17_H_14_O_4_S)_2_(C_10_H_8_N_2_)_2_]	*F*(000) = 1176
*M**_r_* = 1165.85	*D*_x_ = 1.583 Mg m^−^^3^
Monoclinic, *P*2_1_/*c*	Mo *K*α radiation, λ = 0.71073 Å
Hall symbol: -P 2ybc	Cell parameters from 6794 reflections
*a* = 13.567 (3) Å	θ = 2.3–27.8°
*b* = 11.572 (3) Å	µ = 1.02 mm^−^^1^
*c* = 15.595 (4) Å	*T* = 296 K
β = 92.540 (3)°	Block, colorless
*V* = 2446.2 (10) Å^3^	0.35 × 0.34 × 0.32 mm
*Z* = 2	

### Data collection

**Table:**

Bruker SMART BREEZE CCD area-detector diffractometer	5706 independent reflections
Radiation source: fine-focus sealed tube	4170 reflections with *I* > 2σ(*I*)
Graphite monochromator	*R*_int_ = 0.042
phi and ω scans	θ_max_ = 28.2°, θ_min_ = 1.5°
Absorption correction: multi-scan (*SADABS*; Sheldrick, 1996)	*h* = −17→18
*T*_min_ = 0.718, *T*_max_ = 0.737	*k* = −9→15
14971 measured reflections	*l* = −20→20

### Refinement

**Table:**

Refinement on *F*^2^	Primary atom site location: structure-invariant direct methods
Least-squares matrix: full	Secondary atom site location: difference Fourier map
*R*[*F*^2^ > 2σ(*F*^2^)] = 0.036	Hydrogen site location: inferred from neighbouring sites
*wR*(*F*^2^) = 0.090	H-atom parameters constrained
*S* = 1.04	*w* = 1/[σ^2^(*F*_o_^2^) + (0.0222*P*)^2^ + 1.2239*P*] where *P* = (*F*_o_^2^ + 2*F*_c_^2^)/3
5706 reflections	(Δ/σ)_max_ = 0.002
329 parameters	Δρ_max_ = 0.59 e Å^−^^3^
3 restraints	Δρ_min_ = −0.67 e Å^−^^3^

### Special details

**Table:**

Geometry. All esds (except the esd in the dihedral angle between two l.s. planes) are estimated using the full covariance matrix. The cell esds are taken into account individually in the estimation of esds in distances, angles and torsion angles; correlations between esds in cell parameters are only used when they are defined by crystal symmetry. An approximate (isotropic) treatment of cell esds is used for estimating esds involving l.s. planes.
Refinement. Refinement of *F*^2^ against ALL reflections. The weighted *R*-factor *wR* and goodness of fit *S* are based on *F*^2^, conventional *R*-factors *R* are based on *F*, with *F* set to zero for negative *F*^2^. The threshold expression of *F*^2^ > σ(*F*^2^) is used only for calculating *R*-factors(gt) *etc*. and is not relevant to the choice of reflections for refinement. *R*-factors based on *F*^2^ are statistically about twice as large as those based on *F*, and *R*- factors based on ALL data will be even larger.

### Fractional atomic coordinates and isotropic or equivalent isotropic displacement parameters (Å^2^)

**Table:**

	*x*	*y*	*z*	*U*_iso_*/*U*_eq_	Occ. (<1)
Cd1	−0.076611 (16)	0.93279 (2)	0.764046 (13)	0.04782 (9)	
S1	0.27579 (6)	0.85650 (8)	0.73380 (5)	0.0515 (2)	
N1	−0.00382 (18)	0.8216 (2)	0.87232 (14)	0.0398 (6)	
N2	−0.14700 (19)	0.9786 (3)	0.89259 (15)	0.0453 (6)	
O1	−0.17570 (19)	0.8271 (2)	0.67240 (15)	0.0656 (7)	
O2	−0.22891 (18)	1.0016 (3)	0.69612 (15)	0.0637 (7)	
O3	0.06431 (16)	0.9391 (2)	0.68258 (14)	0.0507 (6)	
O4	0.03442 (16)	1.0841 (2)	0.76726 (15)	0.0525 (6)	
C1	0.0686 (2)	0.7475 (3)	0.8589 (2)	0.0514 (8)	
H1	0.0923	0.7415	0.8040	0.062*	
C2	0.1096 (3)	0.6798 (3)	0.9225 (3)	0.0648 (10)	
H2	0.1604	0.6288	0.9113	0.078*	
C3	0.0746 (3)	0.6885 (4)	1.0030 (3)	0.0728 (12)	
H3	0.1007	0.6422	1.0472	0.087*	
C4	0.0007 (3)	0.7660 (3)	1.0185 (2)	0.0603 (10)	
H4	−0.0235	0.7731	1.0731	0.072*	
C5	−0.0371 (2)	0.8332 (3)	0.95146 (17)	0.0397 (7)	
C6	−0.1152 (2)	0.9199 (3)	0.96275 (17)	0.0403 (7)	
C7	−0.1544 (3)	0.9423 (3)	1.0416 (2)	0.0593 (10)	
H7	−0.1325	0.9013	1.0900	0.071*	
C8	−0.2256 (3)	1.0254 (4)	1.0475 (2)	0.0762 (12)	
H8	−0.2527	1.0412	1.1000	0.091*	
C9	−0.2563 (3)	1.0845 (4)	0.9761 (3)	0.0728 (11)	
H9	−0.3044	1.1414	0.9790	0.087*	
C10	−0.2157 (3)	1.0593 (3)	0.9002 (3)	0.0611 (9)	
H10	−0.2369	1.1003	0.8515	0.073*	
C11	−0.2382 (2)	0.9068 (4)	0.66121 (19)	0.0512 (9)	
C12	0.0872 (2)	1.0346 (3)	0.71384 (18)	0.0399 (7)	
C13	0.1804 (2)	1.0957 (3)	0.6894 (2)	0.0527 (8)	
H13A	0.1622	1.1721	0.6684	0.063*	
H13B	0.2218	1.1062	0.7411	0.063*	
C14	0.2415 (2)	1.0395 (3)	0.62373 (19)	0.0390 (7)	
C15	0.2532 (2)	1.0938 (3)	0.5439 (2)	0.0483 (8)	
H15	0.2175	1.1608	0.5313	0.058*	
C16	0.3151 (2)	1.0519 (3)	0.48389 (18)	0.0496 (9)	
C17	0.3342 (3)	1.1070 (4)	0.3955 (2)	0.0409 (10)	0.770 (6)
H17	0.3875	1.0645	0.3690	0.049*	0.770 (6)
C18	0.3637 (5)	1.2319 (5)	0.4048 (4)	0.0575 (15)	0.770 (6)
H18A	0.3749	1.2635	0.3492	0.086*	0.770 (6)
H18B	0.3118	1.2742	0.4306	0.086*	0.770 (6)
H18C	0.4231	1.2377	0.4404	0.086*	0.770 (6)
C17A	0.2979 (9)	1.1532 (9)	0.4175 (6)	0.0409 (10)	0.230 (6)
H17A	0.2655	1.2209	0.4413	0.049*	0.230 (6)
C18A	0.3971 (12)	1.176 (3)	0.3811 (13)	0.079 (8)	0.230 (6)
H18D	0.3915	1.2402	0.3425	0.119*	0.230 (6)
H18E	0.4437	1.1937	0.4273	0.119*	0.230 (6)
H18F	0.4193	1.1091	0.3508	0.119*	0.230 (6)
C19	0.3657 (2)	0.9531 (3)	0.50239 (19)	0.0538 (9)	
H19	0.4095	0.9246	0.4633	0.065*	
C20	0.3535 (2)	0.8944 (3)	0.57788 (19)	0.0470 (7)	
H20	0.3869	0.8252	0.5882	0.056*	
C21	0.2919 (2)	0.9377 (3)	0.63872 (17)	0.0351 (6)	
C22	0.3985 (2)	0.8454 (3)	0.77750 (18)	0.0465 (8)	
C23	0.4312 (3)	0.7405 (3)	0.8089 (2)	0.0577 (9)	
H23	0.3905	0.6758	0.8044	0.069*	
C24	0.5250 (3)	0.7317 (5)	0.8472 (3)	0.0826 (14)	
H24	0.5469	0.6608	0.8687	0.099*	
C25	0.5852 (3)	0.8251 (5)	0.8536 (3)	0.0920 (16)	
H25	0.6480	0.8182	0.8797	0.110*	
C26	0.5534 (4)	0.9301 (4)	0.8216 (3)	0.0930 (17)	
H26	0.5951	0.9939	0.8249	0.112*	
C27	0.4590 (3)	0.9406 (4)	0.7844 (3)	0.0704 (12)	
H27	0.4367	1.0120	0.7642	0.084*	

### Atomic displacement parameters (Å^2^)

**Table:**

	*U*^11^	*U*^22^	*U*^33^	*U*^12^	*U*^13^	*U*^23^
Cd1	0.04260 (13)	0.07524 (19)	0.02515 (11)	−0.00810 (12)	−0.00395 (8)	0.00632 (10)
S1	0.0472 (4)	0.0652 (6)	0.0417 (4)	−0.0015 (4)	−0.0019 (3)	0.0187 (4)
N1	0.0453 (14)	0.0434 (15)	0.0302 (11)	−0.0060 (12)	−0.0031 (10)	−0.0014 (11)
N2	0.0453 (14)	0.0576 (17)	0.0327 (12)	−0.0001 (13)	−0.0010 (10)	0.0031 (12)
O1	0.0723 (17)	0.0725 (19)	0.0504 (13)	−0.0072 (15)	−0.0146 (12)	−0.0019 (13)
O2	0.0636 (16)	0.075 (2)	0.0510 (14)	−0.0048 (14)	−0.0140 (11)	0.0087 (14)
O3	0.0473 (12)	0.0600 (16)	0.0452 (12)	−0.0121 (11)	0.0076 (9)	−0.0100 (11)
O4	0.0461 (12)	0.0600 (16)	0.0519 (13)	0.0043 (11)	0.0093 (10)	−0.0064 (11)
C1	0.056 (2)	0.050 (2)	0.0483 (18)	−0.0058 (16)	0.0004 (15)	−0.0073 (15)
C2	0.062 (2)	0.057 (2)	0.075 (2)	0.0122 (19)	−0.0037 (19)	0.002 (2)
C3	0.078 (3)	0.076 (3)	0.064 (2)	0.009 (2)	−0.010 (2)	0.024 (2)
C4	0.067 (2)	0.079 (3)	0.0349 (16)	−0.001 (2)	−0.0035 (15)	0.0194 (17)
C5	0.0426 (16)	0.0471 (19)	0.0288 (13)	−0.0122 (14)	−0.0056 (11)	0.0016 (12)
C6	0.0414 (15)	0.052 (2)	0.0269 (13)	−0.0128 (14)	−0.0025 (11)	−0.0012 (12)
C7	0.073 (2)	0.073 (3)	0.0320 (15)	0.001 (2)	0.0021 (15)	−0.0047 (16)
C8	0.090 (3)	0.091 (3)	0.048 (2)	0.007 (3)	0.014 (2)	−0.025 (2)
C9	0.077 (3)	0.063 (3)	0.078 (3)	0.012 (2)	0.008 (2)	−0.015 (2)
C10	0.059 (2)	0.062 (3)	0.061 (2)	0.0068 (19)	0.0008 (17)	0.0065 (18)
C11	0.0492 (19)	0.074 (3)	0.0293 (14)	−0.0195 (18)	−0.0064 (13)	0.0211 (16)
C12	0.0363 (15)	0.0480 (19)	0.0350 (14)	0.0045 (13)	−0.0029 (12)	0.0031 (13)
C13	0.0452 (18)	0.046 (2)	0.067 (2)	0.0012 (15)	0.0081 (15)	−0.0095 (16)
C14	0.0284 (13)	0.0426 (18)	0.0454 (16)	−0.0034 (12)	−0.0034 (11)	−0.0021 (13)
C15	0.0338 (15)	0.049 (2)	0.061 (2)	−0.0029 (14)	−0.0176 (14)	0.0174 (16)
C16	0.0398 (16)	0.076 (3)	0.0318 (14)	−0.0213 (17)	−0.0106 (12)	0.0094 (15)
C17	0.031 (2)	0.062 (3)	0.0298 (18)	−0.0041 (19)	0.0038 (14)	0.0031 (18)
C18	0.063 (4)	0.063 (4)	0.046 (3)	−0.020 (3)	−0.003 (2)	0.005 (3)
C17A	0.031 (2)	0.062 (3)	0.0298 (18)	−0.0041 (19)	0.0038 (14)	0.0031 (18)
C18A	0.062 (13)	0.13 (2)	0.050 (12)	−0.021 (14)	−0.007 (9)	0.024 (13)
C19	0.0526 (19)	0.078 (3)	0.0308 (15)	−0.0067 (18)	0.0055 (13)	−0.0073 (16)
C20	0.0495 (18)	0.050 (2)	0.0414 (16)	0.0077 (15)	0.0030 (13)	−0.0021 (15)
C21	0.0359 (14)	0.0401 (17)	0.0290 (13)	0.0008 (13)	−0.0033 (10)	0.0023 (12)
C22	0.0513 (18)	0.057 (2)	0.0312 (14)	0.0008 (16)	−0.0028 (12)	0.0070 (14)
C23	0.062 (2)	0.056 (2)	0.054 (2)	0.0041 (18)	−0.0080 (16)	0.0128 (17)
C24	0.071 (3)	0.102 (4)	0.074 (3)	0.016 (3)	−0.010 (2)	0.037 (3)
C25	0.065 (3)	0.130 (5)	0.078 (3)	−0.007 (3)	−0.027 (2)	0.035 (3)
C26	0.085 (3)	0.105 (4)	0.086 (3)	−0.039 (3)	−0.037 (3)	0.033 (3)
C27	0.076 (3)	0.066 (3)	0.067 (2)	−0.010 (2)	−0.022 (2)	0.023 (2)

### Geometric parameters (Å, º)

**Table:**

Cd1—O1	2.275 (2)	C13—H13A	0.9700
Cd1—O4	2.309 (2)	C13—H13B	0.9700
Cd1—N1	2.310 (2)	C14—C21	1.377 (4)
Cd1—N2	2.320 (3)	C14—C15	1.411 (4)
Cd1—O3	2.343 (2)	C15—C16	1.373 (5)
Cd1—O2	2.415 (2)	C15—H15	0.9300
S1—C22	1.776 (3)	C16—C19	1.359 (5)
S1—C21	1.777 (3)	C16—C17	1.551 (5)
N1—C1	1.328 (4)	C16—C17A	1.574 (9)
N1—C5	1.340 (4)	C17—C18	1.505 (6)
N2—C10	1.329 (4)	C17—C11^i^	1.550 (4)
N2—C6	1.342 (4)	C17—H17	0.9800
O1—C11	1.260 (4)	C18—H18A	0.9600
O2—C11	1.228 (4)	C18—H18B	0.9600
O3—C12	1.242 (4)	C18—H18C	0.9601
O4—C12	1.260 (4)	C17A—C18A	1.506 (10)
C1—C2	1.364 (5)	C17A—C11^i^	1.599 (9)
C1—H1	0.9300	C17A—H17A	0.9800
C2—C3	1.365 (5)	C18A—H18D	0.9600
C2—H2	0.9300	C18A—H18E	0.9600
C3—C4	1.375 (6)	C18A—H18F	0.9600
C3—H3	0.9300	C19—C20	1.375 (5)
C4—C5	1.384 (4)	C19—H19	0.9300
C4—H4	0.9300	C20—C21	1.386 (4)
C5—C6	1.475 (4)	C20—H20	0.9300
C6—C7	1.385 (4)	C22—C23	1.375 (5)
C7—C8	1.369 (6)	C22—C27	1.376 (5)
C7—H7	0.9300	C23—C24	1.385 (5)
C8—C9	1.356 (6)	C23—H23	0.9300
C8—H8	0.9300	C24—C25	1.357 (7)
C9—C10	1.359 (6)	C24—H24	0.9300
C9—H9	0.9300	C25—C26	1.377 (6)
C10—H10	0.9300	C25—H25	0.9300
C11—C17^i^	1.550 (4)	C26—C27	1.387 (6)
C11—C17A^i^	1.599 (9)	C26—H26	0.9300
C12—C13	1.513 (4)	C27—H27	0.9300
C13—C14	1.494 (4)		
			
O1—Cd1—O4	142.02 (9)	C12—C13—H13A	107.9
O1—Cd1—N1	112.41 (9)	C14—C13—H13B	107.9
O4—Cd1—N1	98.58 (8)	C12—C13—H13B	107.9
O1—Cd1—N2	114.29 (10)	H13A—C13—H13B	107.2
O4—Cd1—N2	95.81 (9)	C21—C14—C15	117.2 (3)
N1—Cd1—N2	71.01 (9)	C21—C14—C13	122.9 (3)
O1—Cd1—O3	98.61 (9)	C15—C14—C13	119.9 (3)
O4—Cd1—O3	56.01 (8)	C16—C15—C14	122.7 (3)
N1—Cd1—O3	94.65 (9)	C16—C15—H15	118.7
N2—Cd1—O3	146.99 (8)	C14—C15—H15	118.7
O1—Cd1—O2	55.34 (10)	C19—C16—C15	118.2 (3)
O4—Cd1—O2	107.72 (9)	C19—C16—C17	115.6 (3)
N1—Cd1—O2	146.53 (9)	C15—C16—C17	126.3 (3)
N2—Cd1—O2	85.80 (9)	C19—C16—C17A	145.6 (6)
O3—Cd1—O2	117.05 (8)	C15—C16—C17A	96.2 (6)
C22—S1—C21	102.15 (14)	C18—C17—C11^i^	111.5 (4)
C1—N1—C5	119.5 (3)	C18—C17—C16	111.3 (4)
C1—N1—Cd1	122.7 (2)	C11^i^—C17—C16	107.2 (3)
C5—N1—Cd1	117.8 (2)	C18—C17—H17	109.0
C10—N2—C6	118.9 (3)	C11^i^—C17—H17	108.9
C10—N2—Cd1	123.7 (2)	C16—C17—H17	108.9
C6—N2—Cd1	117.3 (2)	C17—C18—H18A	109.3
C11—O1—Cd1	93.9 (2)	C17—C18—H18B	109.5
C11—O2—Cd1	88.2 (2)	H18A—C18—H18B	109.5
C12—O3—Cd1	90.61 (18)	C17—C18—H18C	109.6
C12—O4—Cd1	91.7 (2)	H18A—C18—H18C	109.5
N1—C1—C2	122.4 (3)	H18B—C18—H18C	109.5
N1—C1—H1	118.8	C18A—C17A—C16	105.6 (13)
C2—C1—H1	118.8	C18A—C17A—C11^i^	102.5 (12)
C1—C2—C3	118.7 (4)	C16—C17A—C11^i^	103.7 (6)
C1—C2—H2	120.6	C18A—C17A—H17A	115.3
C3—C2—H2	120.6	C16—C17A—H17A	113.8
C2—C3—C4	119.6 (3)	C11^i^—C17A—H17A	114.6
C2—C3—H3	120.2	C17A—C18A—H18E	108.9
C4—C3—H3	120.2	H18D—C18A—H18E	109.5
C3—C4—C5	119.0 (3)	C17A—C18A—H18F	110.6
C3—C4—H4	120.5	H18D—C18A—H18F	109.5
C5—C4—H4	120.5	H18E—C18A—H18F	109.5
N1—C5—C4	120.7 (3)	C16—C19—C20	121.2 (3)
N1—C5—C6	116.9 (2)	C16—C19—H19	119.4
C4—C5—C6	122.5 (3)	C20—C19—H19	119.4
N2—C6—C7	120.5 (3)	C19—C20—C21	120.5 (3)
N2—C6—C5	116.9 (3)	C19—C20—H20	119.8
C7—C6—C5	122.6 (3)	C21—C20—H20	119.8
C8—C7—C6	119.4 (3)	C14—C21—C20	120.2 (3)
C8—C7—H7	120.3	C14—C21—S1	121.0 (2)
C6—C7—H7	120.3	C20—C21—S1	118.7 (2)
C9—C8—C7	119.4 (4)	C23—C22—C27	119.8 (3)
C9—C8—H8	120.3	C23—C22—S1	118.9 (3)
C7—C8—H8	120.3	C27—C22—S1	121.2 (3)
C8—C9—C10	119.0 (4)	C22—C23—C24	119.6 (4)
C8—C9—H9	120.5	C22—C23—H23	120.2
C10—C9—H9	120.5	C24—C23—H23	120.2
N2—C10—C9	122.8 (4)	C25—C24—C23	120.9 (4)
N2—C10—H10	118.6	C25—C24—H24	119.6
C9—C10—H10	118.6	C23—C24—H24	119.6
O2—C11—O1	122.5 (3)	C24—C25—C26	119.9 (4)
O2—C11—C17^i^	114.4 (4)	C24—C25—H25	120.1
O1—C11—C17^i^	123.1 (4)	C26—C25—H25	120.1
O2—C11—C17A^i^	140.1 (5)	C25—C26—C27	119.9 (4)
O1—C11—C17A^i^	95.9 (5)	C25—C26—H26	120.1
O3—C12—O4	121.6 (3)	C27—C26—H26	120.1
O3—C12—C13	121.0 (3)	C22—C27—C26	120.0 (4)
O4—C12—C13	117.4 (3)	C22—C27—H27	120.0
C14—C13—C12	117.7 (3)	C26—C27—H27	120.0
C14—C13—H13A	107.9		
			
O1—Cd1—N1—C1	72.7 (3)	C5—C6—C7—C8	178.9 (3)
O4—Cd1—N1—C1	−84.9 (2)	C6—C7—C8—C9	−0.2 (6)
N2—Cd1—N1—C1	−178.1 (3)	C7—C8—C9—C10	0.2 (7)
O3—Cd1—N1—C1	−28.6 (2)	C6—N2—C10—C9	−0.8 (6)
O2—Cd1—N1—C1	133.3 (2)	Cd1—N2—C10—C9	179.7 (3)
O1—Cd1—N1—C5	−107.1 (2)	C8—C9—C10—N2	0.2 (7)
O4—Cd1—N1—C5	95.3 (2)	Cd1—O2—C11—O1	−0.5 (3)
N2—Cd1—N1—C5	2.0 (2)	Cd1—O2—C11—C17^i^	178.0 (2)
O3—Cd1—N1—C5	151.6 (2)	Cd1—O2—C11—C17A^i^	−162.3 (8)
O2—Cd1—N1—C5	−46.6 (3)	Cd1—O1—C11—O2	0.5 (3)
O1—Cd1—N2—C10	−75.4 (3)	Cd1—O1—C11—C17^i^	−177.9 (3)
O4—Cd1—N2—C10	80.8 (3)	Cd1—O1—C11—C17A^i^	168.9 (4)
N1—Cd1—N2—C10	177.9 (3)	Cd1—O3—C12—O4	−0.9 (3)
O3—Cd1—N2—C10	109.8 (3)	Cd1—O3—C12—C13	178.7 (3)
O2—Cd1—N2—C10	−26.6 (3)	Cd1—O4—C12—O3	0.9 (3)
O1—Cd1—N2—C6	105.1 (2)	Cd1—O4—C12—C13	−178.7 (2)
O4—Cd1—N2—C6	−98.7 (2)	O3—C12—C13—C14	2.5 (5)
N1—Cd1—N2—C6	−1.6 (2)	O4—C12—C13—C14	−177.9 (3)
O3—Cd1—N2—C6	−69.7 (3)	C12—C13—C14—C21	−67.9 (4)
O2—Cd1—N2—C6	153.9 (2)	C12—C13—C14—C15	115.3 (3)
O4—Cd1—O1—C11	−73.7 (2)	C21—C14—C15—C16	−2.9 (4)
N1—Cd1—O1—C11	144.02 (19)	C13—C14—C15—C16	174.2 (3)
N2—Cd1—O1—C11	65.6 (2)	C14—C15—C16—C19	0.9 (5)
O3—Cd1—O1—C11	−117.3 (2)	C14—C15—C16—C17	−178.6 (3)
O2—Cd1—O1—C11	−0.26 (18)	C14—C15—C16—C17A	−176.7 (4)
O1—Cd1—O2—C11	0.27 (18)	C19—C16—C17—C18	−126.6 (4)
O4—Cd1—O2—C11	141.99 (19)	C15—C16—C17—C18	53.0 (5)
N1—Cd1—O2—C11	−77.9 (2)	C17A—C16—C17—C18	49.1 (8)
N2—Cd1—O2—C11	−123.2 (2)	C19—C16—C17—C11^i^	111.2 (4)
O3—Cd1—O2—C11	81.7 (2)	C15—C16—C17—C11^i^	−69.2 (5)
O1—Cd1—O3—C12	149.75 (18)	C17A—C16—C17—C11^i^	−73.1 (7)
O4—Cd1—O3—C12	0.51 (16)	C19—C16—C17A—C18A	−34.7 (15)
N1—Cd1—O3—C12	−96.72 (18)	C15—C16—C17A—C18A	141.6 (12)
N2—Cd1—O3—C12	−35.0 (3)	C17—C16—C17A—C18A	−41.6 (13)
O2—Cd1—O3—C12	94.40 (18)	C19—C16—C17A—C11^i^	72.7 (10)
O1—Cd1—O4—C12	−55.7 (2)	C15—C16—C17A—C11^i^	−111.0 (6)
N1—Cd1—O4—C12	89.33 (18)	C17—C16—C17A—C11^i^	65.8 (6)
N2—Cd1—O4—C12	160.94 (18)	C15—C16—C19—C20	1.9 (5)
O3—Cd1—O4—C12	−0.50 (16)	C17—C16—C19—C20	−178.5 (3)
O2—Cd1—O4—C12	−111.62 (18)	C17A—C16—C19—C20	177.7 (7)
C5—N1—C1—C2	1.6 (5)	C16—C19—C20—C21	−2.7 (5)
Cd1—N1—C1—C2	−178.2 (3)	C15—C14—C21—C20	2.0 (4)
N1—C1—C2—C3	0.1 (6)	C13—C14—C21—C20	−174.9 (3)
C1—C2—C3—C4	−1.1 (6)	C15—C14—C21—S1	−175.4 (2)
C2—C3—C4—C5	0.4 (6)	C13—C14—C21—S1	7.6 (4)
C1—N1—C5—C4	−2.4 (4)	C19—C20—C21—C14	0.6 (5)
Cd1—N1—C5—C4	177.5 (2)	C19—C20—C21—S1	178.1 (2)
C1—N1—C5—C6	177.9 (3)	C22—S1—C21—C14	−124.0 (2)
Cd1—N1—C5—C6	−2.3 (3)	C22—S1—C21—C20	58.5 (3)
C3—C4—C5—N1	1.4 (5)	C21—S1—C22—C23	−135.7 (3)
C3—C4—C5—C6	−178.9 (3)	C21—S1—C22—C27	47.8 (3)
C10—N2—C6—C7	0.8 (5)	C27—C22—C23—C24	−0.1 (6)
Cd1—N2—C6—C7	−179.6 (2)	S1—C22—C23—C24	−176.7 (3)
C10—N2—C6—C5	−178.5 (3)	C22—C23—C24—C25	−0.4 (7)
Cd1—N2—C6—C5	1.0 (3)	C23—C24—C25—C26	−0.2 (8)
N1—C5—C6—N2	0.8 (4)	C24—C25—C26—C27	1.3 (8)
C4—C5—C6—N2	−179.0 (3)	C23—C22—C27—C26	1.2 (6)
N1—C5—C6—C7	−178.5 (3)	S1—C22—C27—C26	177.7 (4)
C4—C5—C6—C7	1.7 (5)	C25—C26—C27—C22	−1.8 (8)
N2—C6—C7—C8	−0.4 (5)		

Symmetry code: (i) −*x*, −*y*+2, −*z*+1.

## References

[bb1] Batten, S. R. & Robson, R. (1998). *Angew. Chem. Int. Ed.* **37**, 1460–1494. 10.1002/(SICI)1521-3773(19980619)37:11<1460::AID-ANIE1460>3.0.CO;2-Z 29710936

[bb2] Bruker (2009). *APEX2* and *SAINT* Bruker AXS Inc., Madison, Wisconsin, USA.

[bb3] Lu, J. Y. (2003). *Coord. Chem. Rev.* **246**, 327–347.

[bb4] Moulton, B. & Zaworotko, M. (2001). *Chem. Rev.* **101**, 1629–1658. 10.1021/cr9900432 11709994

[bb5] Pan, L., Sander, M. B., Huang, X., Li, J., Smith, M., Bittner, E., Bockrath, B. & Johnson, J. K. (2004). *J. Am. Chem. Soc.* **126**, 1308–1309. 10.1021/ja0392871 14759166

[bb6] Sheldrick, G. M. (1996). *SADABS* University of Göttingen, Germany.

[bb7] Sheldrick, G. M. (2008). *Acta Cryst.* A**64**, 112–122. 10.1107/S0108767307043930 18156677

[bb8] Wang, X. L., Qin, C., Wang, E. B., Xu, L., Su, Z. M. & Hu, C. W. (2004). *Angew. Chem. Int. Ed.* **43**, 5036–5040. 10.1002/anie.200460758 15384113

